# Cross-Sectional and Longitudinal Assessment of Sociodemographic and Lifestyle Determinants of Metabolic Syndrome and Hypertriglyceridemic Waist Phenotypes in 139,634 Spanish Workers

**DOI:** 10.3390/metabo15070474

**Published:** 2025-07-14

**Authors:** Joan Obrador de Hevia, Ángel Arturo López-González, José Ignacio Ramírez-Manent, Carla Busquets-Cortés, Pedro Juan Tárraga López, Pere Riutord-Sbert

**Affiliations:** 1 Investigation Group ADEMA SALUD, University Institute for Research in Health Sciences (IUNICS), 07010 Palma, Spain; j.obrador@eua.edu.es (J.O.d.H.); joseignacio.ramirez@ibsalut.es (J.I.R.-M.); c.busquets@eua.edu.es (C.B.-C.); p.riutord@eua.edu.es (P.R.-S.); 2 Faculty of Dentistry, University School ADEMA, 07010 Palma, Spain; 3 Institut d’Investigació Sanitària de les Illes Balears (IDISBA), Balearic Islands Health Research Institute Foundation, 07010 Palma, Spain; 4 Primary Care in Mallorca, Balearic Islands Health Service, 07010 Palma, Spain; 5 Faculty of Medicine, University of the Balearic Islands, 07010 Palma, Spain; 6 Faculty of Medicine, University of Castilla La Mancha (UCLM), 02008 Albacete, Spain; pjtarraga@sescam.jccm.es; 7 Health Service of Castilla La Mancha (SESCAM), 02008 Albacete, Spain

**Keywords:** cardiometabolic risk, visceral adiposity, occupational epidemiology, lifestyle, social determinants, metabolic syndrome

## Abstract

**Objective:** The objective of this study was to analyze the prevalence and key sociodemographic and lifestyle determinants of metabolic syndrome (MetS) and the hypertriglyceridemic waist (HTGW) phenotype in a large occupational cohort. **Background:** Metabolic syndrome (MetS) and the hypertriglyceridemic waist (HTGW) phenotype, defined as the simultaneous presence of elevated waist circumference and high triglyceride levels, are major predictors of cardiometabolic morbidity and mortality. Despite their clinical relevance, data on their distribution and determinants in large occupational populations remain limited. **Methods:** A cross-sectional analysis was conducted on 139,634 employed adults (56,352 women and 83,282 men) across Spain, based on standardized clinical evaluations and validated questionnaires assessing physical activity, diet, smoking, alcohol consumption, education, and occupational class. Logistic regression models were used to estimate associations with MetS and HTGW. A longitudinal subsample of 40,431 individuals was followed over a 10-year period (2009–2019) to assess trends in metabolic risk phenotypes. **Results:** Male sex, older age, lower educational attainment, and unhealthy lifestyle behaviors were associated with a higher prevalence of both MetS and the HTGW phenotype. Physical inactivity, low adherence to the Mediterranean diet, and alcohol consumption were significantly associated with increased risk. The HTGW phenotype proved useful in identifying high-risk individuals, with a steadily increasing prevalence over time. **Conclusions:** Sociodemographic disparities and modifiable lifestyle factors significantly influence the prevalence and progression of MetS and HTGW in the Spanish workforce. Preventive strategies should emphasize early workplace screening, promotion of healthy behaviors, and reduction in educational and socioeconomic inequalities to mitigate cardiometabolic risk.

## 1. Introduction

Metabolic syndrome (MetS) represents a constellation of interrelated cardiometabolic risk factors—including central obesity, hypertension, dyslipidemia, and impaired glucose metabolism—that collectively elevate the risk of cardiovascular disease (CVD), type 2 diabetes mellitus (T2DM), and all-cause mortality [1]. The global prevalence of MetS has been steadily increasing, with current estimates indicating that approximately 25% of the adult population is affected [2]. In Spain, the prevalence of MetS varies depending on the diagnostic criteria applied, with studies reporting rates ranging from 24% to 37% [3,4].

The etiology of MetS is multifactorial, encompassing both genetic predispositions and modifiable lifestyle factors such as diet, physical activity, smoking, and alcohol consumption [5]. Socioeconomic status (SES) and educational attainment have also been identified as significant determinants, influencing health-related behaviors and access to healthcare resources [6]. Lower SES and educational levels are consistently associated with a higher prevalence of MetS, likely due to limited health literacy and reduced engagement in preventive health practices [7].

Dietary patterns, particularly adherence to the Mediterranean diet, have been inversely associated with MetS. This dietary pattern, characterized by high consumption of fruits, vegetables, whole grains, legumes, nuts, and olive oil, along with moderate intake of fish and poultry, has demonstrated protective effects against MetS and its components [8]. In contrast, diets high in ultra-processed foods and saturated fats have been linked to an increased risk of MetS [9].

Physical inactivity is another critical modifiable risk factor. Sedentary behavior contributes to obesity, insulin resistance, and dyslipidemia—all key components of MetS [10]. Regular physical activity has been shown to improve metabolic parameters and reduce the incidence of MetS [11].

The hypertriglyceridemic waist (HTGW) phenotype, defined by increased waist circumference and elevated triglyceride levels, has emerged as a practical and cost-effective clinical marker for identifying individuals at high risk for MetS and CVD [12]. This phenotype is particularly prevalent among individuals with poor dietary habits and sedentary lifestyles [13].

Despite a growing body of literature on MetS, large-scale studies investigating the interplay between sociodemographic factors, lifestyle behaviors, and educational attainment within the Spanish working population remain scarce. Understanding these associations is essential for developing targeted public health strategies aimed at reducing the burden of MetS and its complications.

This study aims to address this gap by analyzing data from over 139,000 Spanish workers to explore the relationships between sociodemographic characteristics, lifestyle behaviors, educational attainment, and the prevalence of MetS and the HTGW phenotype. By identifying key determinants, this research seeks to inform the design of effective prevention strategies tailored to high-risk occupational groups.

We hypothesize that lower educational level, unhealthy behaviors, and lower occupational class are significantly associated with increased prevalence of MetS and HTGW in the Spanish working population. The objective is to assess the prevalence and determinants of MetS and HTGW among Spanish workers, with a particular focus on sociodemographic and lifestyle factors. Understanding these associations may support the development of workplace-based preventive interventions and inform national public health policies aimed at mitigating cardiometabolic risk in vulnerable subgroups.

## 2. Methods

### 2.1. Study Design and Participant Selection

The investigation followed a two-stage methodological framework. In the first phase, a cross-sectional descriptive analysis was conducted involving 139,634 employees (56,352 women and 83,282 men) from various autonomous communities in Spain, including the Balearic Islands, Andalusia, Canary Islands, Valencian Community, Catalonia, Madrid, Castilla-La Mancha, Castilla y León, and the Basque Country. Participants represented a broad range of employment sectors, particularly hospitality, commerce, healthcare, public administration, transportation, industry, and cleaning services. Data were collected during routine occupational health assessments conducted by medical units affiliated with the participating companies. The data collection period extended from December 2020 to December 2021.

The second phase comprised a retrospective longitudinal analysis of a subsample of 40,431 individuals (24,229 men and 16,202 women) selected from the original cohort, for whom complete data were available. This phase spanned a ten-year period, from 2009 to 2019.

All anthropometric, clinical, and biochemical data were obtained by trained healthcare professionals employed by the collaborating institutions. Prior to data collection, standardized protocols were implemented to ensure methodological consistency and to minimize inter-observer variability.

### 2.2. Inclusion Criteria

Participants were eligible for inclusion if they met the following criteria:

- Aged between 18 and 69 years, representing the working-age population.
- Actively employed by one of the participating companies and not on medical leave at the time of data collection.
- Availability of complete data required for the calculation of cardiovascular risk metrics.
- Provision of informed consent for the use of anonymized data for epidemiological research.
- For inclusion in the longitudinal analysis: availability of complete records from both 2009 and 2019, with no major changes in sociodemographic or lifestyle characteristics over the study period.

**Figure 1:**
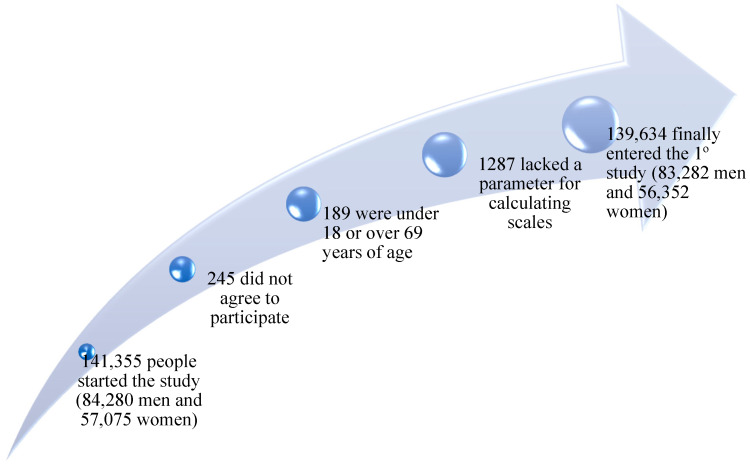
Flowchart of participants.

A flowchart summarizing the recruitment and selection process is provided in Figure 1.

### 2.3. Measurement and Data Collection Procedures

Measurements were conducted by trained professionals following harmonized protocols to ensure accuracy and reproducibility. These procedures included the assessment of body composition, blood pressure, and laboratory biomarkers.

Body weight and height were recorded using a calibrated SECA 700 scale. Waist circumference was measured with a SECA tape, following standard anthropometric procedures: the tape was positioned horizontally at the midpoint between the last palpable rib and the iliac crest, with participants standing upright and relaxed.

Blood pressure was measured using an automatic OMRON M3 device. Readings were taken in a seated position after a minimum of 10 min of rest. Three readings were obtained at one-minute intervals, and the average value was used for analysis [14].

Venous blood samples were collected after at least 12 h of fasting. Laboratory evaluations included plasma glucose, total cholesterol, and triglyceride concentrations, all assessed using enzymatic methods on automated analyzers. HDL-C was measured after precipitation with dextran sulfate–magnesium chloride. LDL-C levels were calculated using the Friedewald equation: LDL = Total cholesterol − HDL − (Triglycerides/5). All biochemical parameters are reported in mg/dL [15].

### 2.4. Definitions of Metabolic Syndrome

#### 2.4.1. NCEP ATP III (National Cholesterol Education Program Adult Treatment Panel III)

MetS is diagnosed when at least three of the following criteria are met:

- Waist circumference > 102 cm in men or >88 cm in women;
- Triglycerides ≥ 150 mg/dL or specific treatment for hypertriglyceridemia;
- Blood pressure ≥ 130/85 mmHg;
- HDL cholesterol < 40 mg/dL in men or <50 mg/dL in women, or specific treatment;
- Fasting glucose > 110 mg/dL or specific treatment for hyperglycemia.

#### 2.4.2. International Diabetes Federation (IDF)

MetS is diagnosed when central obesity is present—defined as a waist circumference > 80 cm in women and >94 cm in men—along with at least two of the following criteria:

- Triglycerides ≥ 150 mg/dL or specific treatment for hypertriglyceridemia;
- Systolic blood pressure ≥ 130 mmHg or diastolic ≥ 85 mmHg, or previous diagnosis of hypertension under treatment;
- HDL cholesterol < 40 mg/dL in men or <50 mg/dL in women, or specific treatment for this lipid abnormality;
- Fasting glucose > 100 mg/dL or previous diagnosis of type 2 diabetes.

#### 2.4.3. Joint Interim Statement (JIS)

MetS is diagnosed when at least three of the following criteria are met:

- Waist circumference > 94 cm in men and > 80 cm in women;
- Triglycerides ≥ 150 mg/dL or specific treatment for hypertriglyceridemia;
- Systolic blood pressure ≥ 130 mmHg or diastolic ≥ 85 mmHg, or previous diagnosis of hypertension under treatment;
- HDL cholesterol < 40 mg/dL in men or <50 mg/dL in women, or specific treatment;
- Fasting glucose > 110 mg/dL or previous diagnosis of type 2 diabetes [16].

As part of the metabolic risk assessment, the hypertriglyceridemic waist (HTGW) phenotype was evaluated based on the concurrent presence of elevated waist circumference and high fasting triglyceride levels. Specifically, individuals were classified as having this phenotype if they exhibited a waist circumference ≥ 102 cm in men or ≥ 88 cm in women, together with fasting triglycerides ≥ 150 mg/dL. This criterion has been validated as a surrogate marker for visceral adiposity and is associated with increased cardiometabolic risk, including insulin resistance and atherogenic dyslipidemia [17]. Identifying this phenotype in occupational cohorts provides a practical and accessible approach to early risk stratification using routine clinical data.

### 2.5. Lifestyle and Sociodemographic Variables

Participants were classified as current smokers if they had smoked at least one cigarette per day (or an equivalent amount of tobacco) in the past month or had quit within the previous year [18].

Diet quality was assessed using the 14-item Mediterranean Diet Adherence Screener (MEDAS), originally developed for the PREDIMED study. Each item scores either 0 or 1, and a total score of ≥9 indicates high adherence to the Mediterranean diet, which is associated with cardiovascular health benefits [19].

Physical activity was evaluated using the International Physical Activity Questionnaire (IPAQ), which captures activity in three domains—occupational, commuting, and leisure—as well as sedentary behavior over the preceding seven days. Activities are categorized as vigorous, moderate, or walking, and results are expressed in MET-minutes/week, a standard metric in public health research [20].

Alcohol consumption was measured in standard alcohol units (SAUs), with one unit equivalent to 10 g of pure ethanol, based on national guidelines. Excessive intake was defined as ≥14 SAUs per week for women and ≥21 SAUs per week for men [21].

Occupational social class was classified according to the 2011 National Classification of Occupations (CNO-11) and criteria established by the Spanish Society of Epidemiology [22]:

- Class I: University-educated professionals and senior executives;
- Class II: Skilled self-employed workers and intermediate-level positions;
- Class III: Manual laborers and unskilled workers.

Educational attainment was categorized into three levels: primary, secondary, and tertiary (university-level) education.

### 2.6. Statistical Methods

Descriptive statistics were used to characterize the study population at baseline. Continuous variables are presented as means and standard deviations (SDs), and group differences were assessed using independent-samples *t*-tests. Categorical variables are presented as percentages, and comparisons were made using chi-square tests.

Multinomial logistic regression models were used to examine the associations between sociodemographic and behavioral factors and the presence of MetS (according to both NCEP ATP III and IDF definitions) and the HTGW phenotype in the cross-sectional dataset. Covariates included age group, sex, educational level, occupational class, smoking status, physical activity, alcohol consumption, and adherence to the Mediterranean diet. Odds ratios (ORs) and 95% confidence intervals (CIs) were calculated for all models.

In addition to the cross-sectional analysis, a retrospective longitudinal analysis was performed on a subsample of 40,431 individuals with complete data from both 2009 and 2019. For this phase, paired comparisons were conducted to evaluate changes in the prevalence of MetS and HTGW over the 10-year period. The percentage change between baseline (PRE) and follow-up (POST) values was calculated using the following formula:

[(POST − PRE)/PRE] × 100

Statistical significance for within-group comparisons was assessed using McNemar’s test for categorical variables. All statistical analyses were conducted using IBM SPSS Statistics version 29.0 (IBM Corp., Armonk, NY, USA). A *p*-value < 0.05 was considered statistically significant for all tests.

## 3. Results

Table 1 and Table 2 provide a detailed sex-stratified overview of anthropometric, biochemical, blood pressure, educational, and lifestyle characteristics among 139,634 Spanish workers. Statistically significant sex differences were observed across nearly all variables (*p* < 0.001), with men exhibiting higher average values for weight, blood pressure, total and LDL cholesterol, triglycerides, and glucose, while women showed higher levels of HDL cholesterol.

**Table 1:** Characteristics of population.

	Men n = 83,282	Women n = 56,352	
	Mean (SD)	Mean (SD)	*p*-Value
Age (years)	41.4 (10.7)	40.1 (10.4)	<0.001
Height (cm)	173.8 (7.1)	161.2 (6.5)	<0.001
Weight (kg)	83.2 (14.6)	66.3 (13.9)	<0.001
Systolic blood pressure (mmHg)	126.2 (15.9)	115.6 (15.7)	<0.001
Diastolic blood pressure (mmHg)	76.6 (10.9)	71.1 (10.7)	<0.001
Total cholesterol (mg/dL)	199.6 (38.6)	194.6 (36.9)	<0.001
HDL cholesterol (mg/dL)	50.0 (7.7)	54.7 (9.2)	<0.001
LDL cholesterol (mg/dL)	122.6 (37.4)	121.5 (37.1)	<0.001
Triglycerides (mg/dL)	133.8 (95.6)	90.8 (49.7)	<0.001
Glucose (mg/dL)	93.0 (25.4)	86.8 (18.1)	<0.001
	%	%	*p*-value
< 30 years	15.1	18.0	<0.001
30–39 years	29.6	31.0	
40–49 years	30.2	30.3	
50–59 years	20.9	17.7	
60–69 years	4.2	3.0	
Social class I	7.5	13.6	<0.001
Social class II	23.8	32.1	
Social class III	68.7	54.1	
Elementary school	66.4	48.1	<0.001
High school	26.9	40.0	
University	6.7	11.9	
Non-smokers	66.8	67.9	<0.001
Smokers	33.2	32.1	
Non-physical activity	62.4	51.4	<0.001
Yes physical activity	37.6	48.6	
Non-Mediterranean diet	65.8	52.8	<0.001
Yes Mediterranean diet	34.2	47.2	
Non-alcohol consumption	67.3	84.4	<0.001
Yes alcohol consumption	32.7	15.6	

LDL—low density lipoprotein-cholesterol. HDL-c—high density lipoprotein-cholesterol. SD—standard deviation.

**Table 2:** Physical characteristics and metabolic indicators of Spanish workers.

	Men n = 83,282	Women n = 56,352	
Variables	Mean (SD)	Mean (SD)	*p*-Value
Age (years)	41.4 (10.7)	40.1 (10.4)	<0.001
Height (cm)	173.8 (7.1)	161.2 (6.5)	<0.001
Weight (kg)	83.2 (14.6)	66.3 (13.9)	<0.001
Systolic blood pressure (mmHg)	126.2 (15.9)	115.6 (15.7)	<0.001
Diastolic blood pressure (mmHg)	76.6 (10.9)	71.1 (10.7)	<0.001
Total cholesterol (mg/dL)	199.6 (38.6)	194.6 (36.9)	<0.001
HDL-cholesterol (mg/dL)	50.0 (7.7)	54.7 (9.2)	<0.001
LDL-cholesterol (mg/dL)	122.6 (37.4)	121.5 (37.1)	<0.001
Triglycerides (mg/dL)	133.8 (95.6)	90.8 (49.7)	<0.001
Glucose (mg/dL)	93.0 (25.4)	86.8 (18.1)	<0.001

HDL—high density lipoprotein. LDL—low density lipoprotein. SD—standard deviation.

Lifestyle-related variables also revealed notable differences: women were more likely to report healthier behaviors, including greater adherence to the Mediterranean diet, higher levels of physical activity, and lower alcohol consumption.

These patterns highlight the importance of sex-disaggregated analysis in research on metabolic and cardiovascular risk, as well as the critical role of social class and educational attainment in shaping health-related behaviors.

Table 3 reveals substantial age- and sex-related gradients in the prevalence of metabolic syndrome (MS) using both ATPIII and IDF definitions. Table 3 presents also substantial age- and sex-related gradients in the prevalence of metabolic syndrome (MetS), based on both ATP III and IDF criteria, as well as the hypertriglyceridemic waist (HTGW) phenotype. Prevalence increases progressively with age in both sexes but remains consistently higher in men across all age groups and socioeconomic strata. For example, the prevalence of MetS-IDF peaks at 35.9% among men aged 60–69, compared to just 14.8% among women in the same age category.

**Table 3:** Prevalence of metabolic syndrome according to NCEP ATP III and IDF criteria, and hypertriglyceridemic waist phenotype (HTGW), with 95% confidence intervals, among Spanish Workers based on sociodemographic, educational, and lifestyle characteristics.

		MS NCEP ATPIII	MS IDF	HTGW
Variables Men	n	% (95% CI)	% (95% CI)	% (95% CI)
<30 years	12,558	3.4 (3.1–3.7)	5.0 (4.6–5.4)	4.7 (4.3–5.1)
30–39 years	24,648	8.8 (8.4–9.2)	11.8 (11.4–12.2)	10.8 (10.4–11.2)
40–49 years	25,178	18.7 (18.2–19.2)	23.2 (22.7–23.7)	18.0 (17.5–18.5)
50–59 years	17,370	29.5 (28.8–30.2)	32.7 (32.0–33.4)	19.0 (18.4–19.6)
60–69 years	3528	44.9 (43.3–46.5)	35.9 (34.3–37.5)	20.1 (18.8–21.4)
Social class I	6234	13.6 (12.8–14.4)	17.1 (16.2–18.0)	10.0 (9.3–10.7)
Social class II	19,856	17.0 (16.4–17.6)	19.0 (18.4–19.6)	14.4 (13.9–14.9)
Social class III	57,192	17.3 (17.0–17.6)	22.2 (21.8–22.6)	15.5 (15.1–15.9)
Elementary school	55,306	20.9 (20.6–21.2)	22.8 (22.5–23.1)	14.3 (14.0–14.6)
High school	22,408	15.4 (15.0–15.8)	18.5 (18.1–18.9)	15.5 (15.1–15.9)
University	5568	14.5 (13.7–15.3)	18.0 (17.1–18.9)	10.3 (9.6–11.0)
Non-smokers	55,618	15.8 (15.5–16.1)	19.4 (19.1–19.7)	14.2 (13.9–14.5)
Smokers	27,664	19.0 (18.5–19.5)	19.7 (19.2–20.2)	14.6 (14.2–15.0)
Non-physical activity	51,984	26.4 (26.0–26.8)	30.6 (30.2–31.0)	23.0 (22.6–23.4)
Yes physical activity	31,298	0.9 (0.7–1.1)	1.4 (1.2–1.6)	2.3 (2.0–2.6)
Non-Mediterranean diet	54,792	25.0 (24.6–25.4)	28.9 (28.5–29.3)	21.8 (21.4–22.2)
Yes Mediterranean diet	28,490	1.2 (1.0–1.4)	1.7 (1.5–1.9)	3.4 (3.1–3.7)
Non-alcohol consumption	56,022	7.6 (7.3–7.9)	8.8 (8.5–9.1)	6.1 (5.8–6.4)
Yes alcohol consumption	27,260	35.8 (35.1–36.5)	41.8 (41.1–42.5)	31.3 (30.6–32.0)
**Variables Women**	**n**	**% (95% CI)**	**% (95% CI)**	**% (95% CI)**
<30 years	10,110	1.4 (1.2–1.6)	1.2 (1.0–1.4)	0.9 (0.7–1.1)
30–39 years	17,460	3.4 (3.1–3.7)	3.0 (2.7–3.3)	1.6 (1.4–1.8)
40–49 years	17,094	8.3 (7.9–8.7)	6.5 (6.1–6.9)	2.9 (2.6–3.2)
50–59 years	9984	18.5 (17.7–19.3)	12.0 (11.4–12.6)	5.5 (5.1–5.9)
60–69 years	1704	29.0 (26.8–31.2)	14.8 (13.1–16.5)	6.1 (5.0–7.2)
Social class I	7632	3.3 (2.9–3.7)	2.7 (2.3–3.1)	1.3 (1.0–1.6)
Social class II	18,112	7.7 (7.3–8.1)	6.1 (5.7–6.5)	2.7 (2.4–3.0)
Social class III	30,608	9.3 (9.0–9.6)	6.8 (6.5–7.1)	3.1 (2.9–3.3)
Elementary school	27,086	9.6 (9.3–9.9)	6.5 (6.2–6.8)	3.2 (3.0–3.4)
High school	22,574	7.3 (7.0–7.6)	5.6 (5.3–5.9)	2.8 (2.6–3.0)
University	6692	3.4 (3.0–3.8)	2.8 (2.4–3.2)	1.0 (0.8–1.2)
Non-smokers	38,252	7.1 (6.8–7.4)	4.9 (4.6–5.2)	2.7 (2.5–2.9)
Smokers	18,100	8.4 (7.9–8.9)	6.0 (5.6–6.4)	2.8 (2.5–3.1)
Non-physical activity	28,962	15.2 (14.7–15.7)	11.1 (10.6–11.6)	5.3 (5.0–5.6)
Yes physical activity	27,390	0.3 (0.2–0.4)	0.7 (0.5–0.9)	0.5 (0.3–0.7)
Non-Mediterranean diet	29,764	14.4 (13.9–14.9)	10.7 (10.2–11.2)	5.2 (4.9–5.5)
Yes Mediterranean diet	26,588	0.4 (0.3–0.5)	1.2 (1.0–1.4)	0.9 (0.7–1.1)
Non-alcohol consumption	47,536	2.6 (2.4–2.8)	1.9 (1.7–2.1)	1.1 (0.9–1.3)
Yes alcohol consumption	8816	16.8 (16.0–17.6)	16.6 (15.8–17.4)	11.9 (11.2–12.6)

MS—metabolic syndrome. NCEP ATPIII—National Cholesterol Education Program Adult Treatment. Panel III. IDF—International Diabetes Federation. HTGW—hypertriglyceridemic waist.

Unhealthy lifestyle behaviors—particularly alcohol consumption and physical inactivity—are strongly associated with higher prevalence rates of MetS and HTGW, especially among men. In contrast, individuals reporting adherence to the Mediterranean diet exhibit substantially lower prevalence across all metabolic phenotypes.

Table 4 presents the adjusted odds ratios (ORs) for the presence of MetS-ATPIII, MetS-IDF, and the hypertriglyceridemic waist (HTGW) phenotype across a range of sociodemographic and lifestyle factors. Male sex, older age, lower educational attainment, lower occupational class, smoking, physical inactivity, low adherence to the Mediterranean diet, and alcohol consumption all emerged as significant and independent risk factors for all three outcomes.

**Table 4:** Adjusted prevalence estimates with 95% confidence intervals derived from logistic regression models based on sociodemographic, educational, and lifestyle characteristics in Spanish workers.

	MS NCEP ATPIII	MS IDF	HTGW
Variables	OR (95% CI)	OR (95% CI)	OR (95% CI)
Women	1	1	1
Men	1.61 (1.54–1.67)	3.05 (2.92–3.19)	4.25 (4.01–4.50)
<30 years	1	1	1
30–39 years	1.87 (1.75–1.99)	1.14 (1.09–1.24)	1.23 (1.16–1.30)
40–49 years	2.70 (2.51–2.90)	1.22 (1.17–1.28)	1.77 (1.56–1.97)
50–59 years	4.38 (4.05–4.72)	1.86 (1.72–2.01)	2.23 (1.88–2.59)
60–69 years	7.90 (7.09–8.70)	3.17 (2.86–3.49)	3.42 (2.60–4.23)
Social class I	1	1	1
Social class II	1.21 (1.15–1.27)	1.19 (1.15–1.23)	1.19 (1.13–1.25)
Social class III	1.93 (1.74–2.13)	1.63 (1.49–1.77)	1.88 (1.61–2.15)
University	1	1	1
High school	1.25 (1.18–1.32)	1.23 (1.18–1.28)	1.14 (1.09–1.19)
Elementary school	2.05 (1.80–2.30)	1.59 (1.50–1.69)	2.15 (1.88–2.42)
Non-smokers	1	1	1
Smokers	1.23 (1.18–1.29)	1.25 (1.18–1.32)	1.19 (1.14–1.25)
Yes physical activity	1	1	1
Non-physical activity	10.50 (9.07–11.94)	9.92 (8.61–11.23)	12.33 (10.12–12.53)
Yes Mediterranean diet	1	1	1
Non-Mediterranean diet	2.18 (1.91–2.46)	2.07 (1.81–2.34)	7.35 (6.03–8.67)
Non-alcohol consumption	1	1	1
Yes alcohol consumption	4.53(4.35–4.72)	4.52 (4.34–4.71)	4.31 (4.12–4.51)

MS Metabolic syndrome. NCEP ATPIII National Cholesterol Education Program Adult Treatment. Panel III. IDF International Diabetes Federation. HTGW Hypertriglyceridemic waist.

Notably, the associations were particularly strong for physical inactivity (e.g., OR = 10.50 for MetS-ATPIII) and poor adherence to the Mediterranean diet (e.g., OR = 7.35 for HTGW), highlighting the critical role of modifiable lifestyle behaviors in cardiometabolic risk.

Table 5 summarizes the findings of the retrospective longitudinal analysis conducted on a subsample of 40,431 Spanish workers over a 10-year period (2009–2019). The table reports changes in the prevalence of metabolic syndrome (MetS) based on both NCEP-ATPIII and IDF criteria, as well as the hypertriglyceridemic waist (HTGW) phenotype, disaggregated by sex and stratified by age, occupational class, educational level, smoking status, physical activity, dietary habits, and alcohol consumption.

**Table 5:** Prevalence of metabolic syndrome according to NCEP ATP III and IDF criteria, and hypertriglyceridemic waist phenotype (HTGW), before and after the study period, with absolute differences, stratified by sex and sociodemographic, educational, and lifestyle characteristics.

			MS NCEP ATPIII			MS IDF			HTGW	
		PRE	POST		PRE	POST		PRE	POST	
Variables Men	n	%	%	Difference (%)	%	%	Difference (%)	%	%	Difference (%)
<30 years	3645	3.3	3.5	4.8	4.6	4.8	5.1	4.5	4.6	2.8
30–39 years	6933	8.2	9.0	9.8	11.0	12.1	9.6	10.0	10.7	6.9
40–49 years	7013	15.8	17.6	11.3	20.7	22.9	10.8	16.1	17.8	10.8
50–59 years	4952	22.9	27.8	21.2	26.6	31.9	20.1	16.3	19.2	17.8
Social class I	1760	7.8	8.5	8.5	9.7	10.2	5.2	5.5	5.8	5.6
Social class II	5368	11.8	13.2	11.8	15.4	16.8	8.8	9.7	10.6	9.5
Social class III	15,415	14.9	17.4	16.9	22.7	25.9	13.9	16.8	19.9	18.2
Elementary school	14,914	14.5	17.0	17.0	21.8	24.8	14.0	16.5	19.5	18.0
High school	6053	11.6	13.0	12.0	16.3	17.7	8.7	10.2	11.2	9.6
University	1576	7.6	8.2	8.3	10.0	10.5	5.3	5.7	6.0	5.7
Non-smokers	15,122	11.6	12.5	8.0	13.9	14.8	6.2	9.4	10.1	7.5
Smokers	7421	16.9	19.8	17.2	20.7	23.8	14.8	17.7	19.9	12.4
Yes physical activity	8535	4.4	4.6	5.0	6.3	6.5	3.3	4.9	5.1	3.3
Non-physical activity	14,008	27.1	31.9	17.9	22.4	25.9	15.5	20.1	23.4	16.2
Yes Mediterranean diet	7767	6.1	6.5	5.7	7.6	7.9	3.9	6.6	6.9	3.9
Non-Mediterranean diet	14,776	25.7	29.8	16.1	18.3	20.9	14.1	16.3	18.8	15.1
Non-alcohol consumption	15,107	8.4	8.8	4.5	9.5	10.2	6.9	9.6	10.3	6.8
Yes alcohol consumption	7436	18.3	21.9	19.5	22.5	24.9	10.9	20.7	24.3	17.3
**Variables Women**	**n**	**%**	**%**	**Difference (%)**	**%**	**%**	**Difference (%)**	**%**	**%**	**Difference (%)**
<30 years	2833	1.6	1.6	2.9	1.5	1.5	3.1	0.9	0.9	1.9
30–39 years	4824	3.1	3.3	4.9	2.9	3.1	5.1	1.6	1.7	5.1
40–49 years	4636	7.5	8.2	8.8	5.9	6.4	9.0	2.8	3.0	9.0
50–59 years	2768	15.9	17.9	12.6	10.6	11.9	12.5	5.1	5.7	11.8
Social class I	1973	5.5	5.9	6.8	4.2	4.4	4.7	1.7	1.8	4.4
Social class II	4920	7.6	8.2	8.5	5.7	6.1	7.9	2.7	2.9	6.9
Social class III	8168	14.2	15.8	11.2	9.9	11.2	13.1	5.2	5.8	12.0
Elementary school	7289	13.4	14.9	11.0	9.6	10.8	12.8	5.1	5.7	11.8
High school	6056	8.4	9.1	8.7	6.2	6.7	8.1	2.9	3.1	7.1
University	1716	5.8	6.2	6.9	4.3	4.5	4.8	1.8	1.9	4.5
Non-smokers	10,236	7.5	7.9	5.5	5.5	5.8	6.0	2.5	2.6	5.5
Smokers	4825	10.7	11.8	10.2	8.6	9.5	10.9	4.4	4.8	9.9
Yes physical activity	7317	3.0	3.1	3.1	2.3	2.4	2.9	1.1	1.1	1.9
Non-physical activity	7744	14.2	16.2	14	12.9	14.5	12.5	5.9	6.5	10.8
Yes Mediterranean diet	7029	3.8	3.9	3.9	2.8	2.9	3.5	1.6	1.6	2.5
Non-Mediterranean diet	8032	13.1	14.8	13.1	11.7	13.0	11.0	5.3	5.8	9.1
Non-alcohol consumption	12,750	6.2	6.5	5.0	4.6	4.8	5.0	2.0	2.1	4.8
Yes alcohol consumption	2311	12.0	13.3	10.9	8.3	9.2	10.5	4.9	5.4	10.9

MS—metabolic syndrome. NCEP ATPIII—National Cholesterol Education Program Adult Treatment Panel III. IDF—International Diabetes Federation. HTGW—hypertriglyceridemic waist. PRE = year 2009, POST = year 2019. The formula for calculating the difference is [POST − PRE/PRE] as a percentage.

The results reveal a consistent upward trend in the prevalence of all three conditions over the decade, particularly among men, individuals with lower educational attainment, and those engaging in unhealthy behaviors. For example, among men aged 50–59, the prevalence of MetS-IDF increased from 26.6% to 31.9%, while HTGW rose from 16.3% to 19.2%. Among women with only primary education, MetS-IDF prevalence rose from 9.6% to 10.8%, and HTGW from 5.1% to 5.7%. These changes reinforce the patterns observed in the cross-sectional data and suggest a progressive deterioration in cardiometabolic profiles over time.

The most pronounced relative increases were observed among individuals who were physically inactive or who did not adhere to the Mediterranean diet. For instance, HTGW prevalence in physically inactive men rose from 20.1% to 23.4%, while MetS-IDF prevalence among non-adherent men increased from 18.3% to 20.9%. These trends highlight the cumulative adverse impact of unhealthy behaviors on metabolic health and underscore the urgent need for sustained public health interventions.

Overall, Table 5 strengthens the evidence that lifestyle factors and social determinants not only influence current metabolic risk but also shape long-term health trajectories. The longitudinal component of the study adds valuable insight into the progression of cardiometabolic risk and suggests potential reversibility through targeted, behavior-based interventions.

## 4. Discussion

This large-scale study underscores the critical influence of sociodemographic, lifestyle, and educational variables on the prevalence of metabolic syndrome (MetS) and the hypertriglyceridemic waist (HTGW) phenotype within a representative cohort of Spanish workers. The findings reveal significant disparities by sex, age, educational attainment, and health-related behaviors, consistent with prior research and reinforcing the urgency of targeted interventions.

Low-grade chronic inflammation has been identified as a central mechanism in the pathophysiology of MetS and its association with hypertriglyceridemia [23]. Visceral obesity and insulin resistance contribute to a pro-inflammatory state in which hypertrophied adipocytes and infiltrating immune cells secrete cytokines such as TNF-α, IL-6, and MCP-1. These mediators impair insulin signaling and promote lipolysis, increasing the flux of free fatty acids to the liver and stimulating hepatic triglyceride synthesis and very-low-density lipoprotein (VLDL) production. These triglyceride-rich lipoproteins exacerbate dyslipidemia and sustain systemic inflammation [24].

Additionally, hypertriglyceridemia has been positively associated with elevated levels of high-sensitivity C-reactive protein (hsCRP), functioning as both a marker and an amplifier of the inflammatory state in MetS. This relationship supports its role in the development of atherosclerosis and type 2 diabetes, emphasizing the need for comprehensive clinical management [25].

The substantially higher prevalence of MetS and HTGW among men aligns with findings from European and global cohorts [26], including data from the European Health Interview Survey and the European Prospective Investigation into Cancer and Nutrition (EPIC) study, both of which linked male sex and older age with increased cardiometabolic risk [27].

This study also highlights strong associations between lower educational attainment and greater odds of both MetS and HTGW, underscoring the persistent impact of social determinants on health. These disparities may be partially mediated by differences in health literacy, healthcare access, and adoption of preventive behaviors [28].

However, it should be noted that data were collected exclusively from an actively employed population, potentially underestimating the true burden of MetS and HTGW relative to the general population. Individuals with severe health conditions or unemployed individuals, who may exhibit higher prevalence rates, were excluded. This selection bias has been acknowledged and addressed in the study’s limitations.

Adherence to the Mediterranean diet emerged as a significant protective factor, consistent with findings from the PREDIMED trials and the SUN cohort, where better diet quality was inversely associated with visceral adiposity and metabolic dysfunction [29,30]. Similarly, regular physical activity was independently associated with reduced odds of MetS and HTGW, underscoring the value of structured workplace wellness programs. A recent meta-analysis confirmed the dose-dependent benefits of physical activity in reducing cardiometabolic risk [31].

Of particular concern, physical inactivity and low adherence to the Mediterranean diet showed the highest odds ratios for all outcomes studied. This pattern reflects the clustering of unhealthy behaviors observed in the Global Burden of Disease (GBD) Study and the UK Biobank [32,33]. Furthermore, alcohol consumption—especially among men—was strongly associated with elevated risk, despite ongoing debate about threshold effects and potential benefits of light-to-moderate intake [34].

A key limitation is the cross-sectional design, which precludes causal inference and raises the possibility of reverse causality. While associations were found between lifestyle factors and MetS, it is plausible that some behaviors (e.g., poor diet or reduced activity) may result from, rather than cause, metabolic conditions. Moreover, self-reported lifestyle data, although collected via validated instruments, are vulnerable to recall and social desirability biases.

Despite these limitations, the HTGW phenotype proved to be a sensitive and practical surrogate marker for visceral adiposity and insulin resistance, as supported by previous occupational studies [35,36]. Its simplicity and reliance on routine clinical measurements enhance its utility in both primary and occupational care.

The consistency and magnitude of associations between lifestyle behaviors and the prevalence of MetS and HTGW reinforce the critical role of modifiable factors in the etiology of these metabolic conditions. Unhealthy behaviors sustained over time appear to have a cumulative impact on metabolic deterioration, significantly increasing cardiometabolic disease risk.

Importantly, the retrospective longitudinal analysis of 40,431 workers adds temporal depth to these findings, demonstrating a progressive worsening of cardiometabolic risk over a 10-year period (2009–2019). Rising prevalence of MetS and HTGW across all subgroups—particularly men, older adults, and individuals with lower educational attainment—mirrors trends observed in other long-term population-based studies, such as the Spanish PREDAPS cohort [37] and the Framingham Offspring Study [38].

The longitudinal data also highlight the impact of persistent unhealthy behaviors. For example, HTGW prevalence in physically inactive men rose from 20.1% to 23.4%, consistent with patterns observed in the AusDiab and D.E.S.I.R. studies, which documented the cumulative effect of sedentary lifestyles on metabolic deterioration [39,40]. Likewise, workers with only primary education experienced the greatest increases in MetS and HTGW, replicating the educational gradient reported in the Whitehall II Study and the EPIC-Norfolk cohort [41,42].

These comparisons strengthen the external validity of our findings and suggest that the deterioration of metabolic health among Spanish workers reflects a broader international trend. Moreover, they reaffirm the relevance of the HTGW phenotype as a simple, early marker for metabolic risk in occupational settings.

From a public health perspective, the data underscore how lifestyle and social determinants shape both current and future metabolic trajectories. While metabolic alterations may follow a progressive course, they may still be reversible with sustained, targeted interventions.

## 5. Strengths

- Large sample size: With over 139,000 participants and a longitudinal subsample exceeding 40,000, the study offers high statistical power and subgroup granularity.
- Occupational population: Inclusion of actively employed individuals facilitates development of pragmatic, workplace-based health promotion policies.
- Integrated phenotyping: Simultaneous assessment of MetS using both ATP III and IDF criteria, along with HTGW, enhances diagnostic robustness.
- Comprehensive variable set: Inclusion of sociodemographic, clinical, lifestyle, and dietary data enables multidimensional analyses.
- Temporal perspective: The 10-year retrospective follow-up provides insight into trends and trajectories of metabolic risk.
- Validated instruments: The use of validated tools (e.g., MEDAS and IPAQ) ensures reliable assessment of key behavioral variables in both cross-sectional and longitudinal contexts.

## 6. Limitations

- Healthy worker effect: Restricting the sample to actively employed individuals likely underestimates the true burden of metabolic disorders in the general population [43].
- Cross-sectional design: Limits causal inference and raises concerns about reverse causality (e.g., metabolic conditions leading to poor lifestyle choices). While longitudinal data were included, the retrospective design and lack of intermediate time points limit causal modeling.
- Self-reported lifestyle data: Despite validated instruments, responses may be influenced by recall or social desirability biases, particularly in a working adult population [44].
- Omission of psychosocial variables: Important factors such as stress, sleep patterns, and chronotype were not assessed, although they are increasingly recognized as relevant to metabolic risk [45].

## 7. Conclusions

In conclusion, this study highlights the substantial prevalence of metabolic syndrome (MetS) and the hypertriglyceridemic waist (HTGW) phenotype among Spanish workers, particularly among men, individuals with lower educational attainment, and those engaging in unhealthy lifestyle behaviors. The strength and consistency of the associations with modifiable risk factors underscore the critical need for preventive strategies tailored to these high-risk groups. Implementing targeted interventions within occupational settings offers a promising avenue to promote healthier behaviors and reduce cardiometabolic risk at the population level.

Future longitudinal research is warranted to elucidate causal mechanisms and rigorously assess the long-term effectiveness of workplace-based preventive interventions.

## Data Availability

The dataset generated and analyzed during the current study is stored in a secure database managed by ADEMA-Escuela Universitaria, in compliance with data protection regulations. The designated Data Protection Officer is Ángel Arturo López González.
